# Real-Time Vibration Feedback from a Smartphone Application Reduces Sedentary Time but Does Not Increase Physical Activity Among Medical Students

**DOI:** 10.3390/healthcare12212133

**Published:** 2024-10-26

**Authors:** Ahmed M. Wafi, Mohammed A. Zaeri, Abdullah A. Khudier, Abdullah N. Abushara, Muath M. Adawi, Luay A. Zakri, Mohammed H. Madkhali, Abdullah S. Al Othaymeen, Wasan M. Qawfashi, Raghad M. Alnami, Anas E. Ahmed

**Affiliations:** 1 Basic Medical Science Department, Faculty of Medicine, Jazan University, Jazan 45142, Saudi Arabia; 2 Faculty of Medicine, Jazan University, Jazan 45142, Saudi Arabia; 3 Family and Community Medicine Department, Faculty of Medicine, Jazan University, Jazan 45142, Saudi Arabia; aeahmed@jazanu.edu.sa

**Keywords:** sedentary behavior, mobile health applications (mhealth apps), vibration feedback, medical students, physical activity

## Abstract

**Background:** Sedentary behavior is associated with various adverse health outcomes. Medical students often experience high academic demands, leading to increased sedentary time. This study aimed to evaluate the effectiveness of a mobile app providing real-time feedback in reducing total sedentary time and prolonged sedentary bouts and in promoting physical activity among medical students. **Methods:** Seventy-seven medical students from Jazan University (mean age: 21.4 years; range: 20–25 years) participated in this study. Participants were assigned to either the control group (*n* = 40) or the intervention group (*n* = 37). The intervention group received real-time vibration feedback via a mobile app, prompting movement every 30 min of sedentary time, while the control group received no intervention. Sedentary behavior and physical activity levels were assessed using the Activities Completed Over Time in 24 h. Paired t-tests were conducted to examine within-group changes, and a two-way ANOVA was used to assess the interaction effect of time and group on sedentary time and physical activity. **Results:** After six weeks, the intervention group showed significant within-group reductions in their total sedentary time of 1.82 h (*p* = 0.01) and prolonged sedentary bouts of 1.91 h (*p* = 0.001), while the control group had no significant changes. Physical activity levels did not significantly change within either group. The two-way ANOVA revealed that there was no significant change over time between the two groups in their total sedentary time F (1, 75) = 1.590, *p* = 0.21, prolonged sedentary bouts F (1, 75) = 3.499, *p* = 0.06, or physical activity F (1, 75) = 0.565, *p* = 0.45. **Conclusions:** Real-time feedback from mobile apps resulted in significant within-group reductions in total and prolonged sedentary time among medical students in the intervention group. Low-cost mobile apps providing real-time feedback may be an effective intervention for reducing sedentary behavior among medical students, potentially improving their health and well-being.

## 1. Introduction

Sedentary behavior (SB) is defined as any waking behavior characterized by an energy expenditure of ≤1.5 metabolic equivalents while in a sitting, reclining, or lying posture [1]. Prolonged SB is associated with numerous adverse health outcomes, including cardiovascular diseases, mental health issues, and increased rates of all-cause mortality [1,2,3]. Importantly, the relationship between SB and all-cause mortality is influenced by the amount of moderate-to-vigorous physical activity (MVPA) individuals engage in.

Evidence suggests that the detrimental effects of prolonged sitting are more pronounced among those with lower levels of MVPA. Biswas et al. conducted a meta-analysis, revealing that individuals with high sedentary time and low physical activity (PA) levels had a significantly higher risk of all-cause mortality compared to those with high PA levels [1]. Furthermore, higher amounts of MVPA attenuated the association between SB and all-cause mortality [4,5]. These findings suggest that while high levels of PA can mitigate some of the risks associated with prolonged sitting, reducing sedentary time remains crucial, especially for those who are less active.

Moreover, the pattern in which SB is accumulated also predicts adverse health outcomes. While insufficient evidence is available to determine whether breaks in sedentary time are important factors in the relationship between SB and cardiovascular disease mortality [6], regularly interrupting extended periods of sitting with short breaks has been shown to improve cardiometabolic health [7,8]. Thus, breaking up prolonged sitting may be beneficial for mitigating many of the deleterious health consequences associated with high levels of SB.

In recent years, there has been a growing interest in exploring how technological devices, especially mobile phones, affect health-related behaviors such as SB. Mobile health (mhealth) applications have emerged as promising tools to encourage healthy lifestyles by promoting PA and reducing SB [9]. Smartphones offer the potential to help users decrease sedentary time by tracking activity levels and providing immediate feedback on their current status. For example, an intervention study involving 58 Belgian adults demonstrated that providing feedback on sedentary habits helped participants decrease their SB [10].

Medical students, due to their rigorous academic commitments, are particularly susceptible to high levels of SB. Prolonged periods of sitting during lectures and study sessions contribute to habitual sedentary behavior, often leading students to be unaware of the extent of their sedentary time. Indeed, students tend to underestimate their daily sedentary time by as much as 2–4 h [11,12]. Reducing SB is particularly challenging for medical students; therefore, interventions that integrate seamlessly into their daily routines, such as vibration reminders from mobile fitness apps, may be effective in reducing SB [9].

Various interventions have been implemented to reduce SB among university students, such as portable pedal machines [13], sanding disks [14], and standing desks with decisional cues [15]. While these interventions appear to decrease SB in university settings, they may be costly and are specific to one domain. In addition, some studies have utilized text messaging to target the SB of university students [16,17]; although this approach enables the intervention to reach a large number of participants, it does not provide responsive intervention delivery, for instance, it does not adapt the timing of the alerts based on the user’s behavior. Furthermore, studies aiming to reduce SB have often utilized accelerometers to measure SB and PA [18,19]. While accelerometers provide objective measurements, their use can be costly, especially in resource-constrained settings. Self-report tools like the 24 h Physical Activity Recall (ACT24) offer a practical alternative for assessing changes in sedentary time and PA in response to mhealth applications.

Given the abovementioned limitations, the primary purpose of this study was to examine whether real-time vibration feedback can reduce total sedentary time and prolonged sedentary bouts. The secondary objective was to examine the effect of this intervention on PA. We hypothesize that medical students who receive vibration feedback reminders from a mobile app will have lower sedentary times than those who do not receive such feedback.

## 2. Method

### 2.1. Participants

Seventy-seven male and female medical students from Jazan University were willing to participate and were recruited between April and May 2024. The participants were assigned to either the control group (C) (*n* = 40) or the sedentary feedback (SF) group (*n* = 37). The inclusion criteria for the study that participants were full-time students between the ages of 18 and 26 years, had no mobility limitations, and were able to use a smartphone. Participants were recruited using stratified sampling to enroll an approximately equal number of males and females and an equal number from each study year (e.g., 2nd year, 3rd year, etc.).

### 2.2. Study Design

Ethical approval for the study was obtained from the Standing Committee for Scientific Research at Jazan University (approval number REC-45/05/864) on 4 December 2023. All participants provided informed consent before their participation. The study was designed as a single-blind randomized controlled trial. Participants reported their sedentary time and PA using the Activities Completed Over Time in 24 h (ACT24), a web-based self-administered instrument, at baseline and again after six weeks of the intervention. Both the participants and the staff responsible for providing instructions were aware of the group assignments; however, the personnel involved in data entry and the quality control of the ACT24 data were blinded to group assignments.

**Figure 1:**
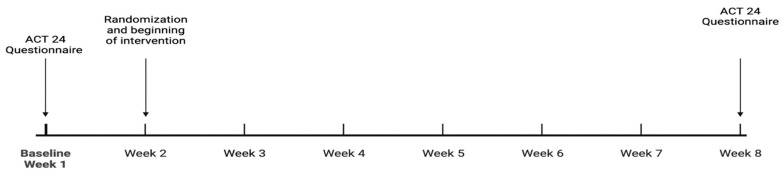
The study design.

The study design is outlined in Figure 1. The protocol involved two laboratory visits: one at baseline and another at week 2. During the first visit, study personnel explained to the students how to access and complete the ACT24 questionnaire. At the second visit, held during week 2, students received minimal education regarding the risks of a sedentary lifestyle, such as the increased risk of cardiac and metabolic disease, as young adults may be unaware of the health implications of sedentary time.

Following the second visit, participants were allocated to either the C group or SF group through stratified random sampling. Those in the SF group had the “Stand Up! The Work Break Timer” app installed on their smartphones, and they were informed that they would receive real-time feedback in the form of vibrations from the app every 30 min during waking hours. These participants were instructed to move in whatever way was feasible at the time of the alert (e.g., stand up or take a walk) or as soon as possible if breaking up their sitting time was not practical (e.g., during class or while driving).

Participants in the C group did not have the app installed on their phones and did not receive vibration reminders to break their SB. To provide a more standardized assessment of the intervention’s impact and ensure consistency in reporting SB and PA, all participants were instructed to complete and submit their recalls on a weekday at baseline and six weeks post-intervention.

### 2.3. Sedentary Time and Physical Activity

Sedentary behavior and PA levels were assessed using the ACT24. The ACT24 is a validated self-report tool that captures detailed information on the types and durations of activities performed over a 24 h period. The ACT24 instrument has been validated for estimating active and sedentary time at the group level. A study comparing the ACT24 with the activPAL device, a thigh-worn accelerometer, found that ACT24 accurately estimates sedentary and active time, with correlations of R = 0.61 (95% CI: [0.39, 0.76]) for sedentary time and R = 0.65 (95% CI: [0.44, 0.78]) for PA time [20]. Participants were instructed to complete the ACT24 questionnaire at baseline and at the end of the intervention period.

Participants reported how much time they spent sleeping, being physically active, and performing SB on the previous day (midnight to midnight). They recorded each activity’s start and end times, allowing for the precise calculation of total sedentary time and PA levels. Individual activities were classified according to six major domains: personal care, leisure, work, household, transportation, and others (spiritual pursuits and private time). For the purpose of this study, participants were instructed to enter the time spent in school or carrying out educational activities under the work domain.

Recalls with a minimum of three activities and at least 22 h of information were considered valid and included in the analysis. To handle recalls with overlapping activities (more than one activity reported at the same time), the activity classified as active was prioritized over sedentary or sleeping activities.

To calculate sedentary bouts of more than 30 min, reported sedentary times that lasted for 30 consecutive minutes or more were identified, and their durations were added for each participant.

### 2.4. Statistical Analysis

To determine the appropriate sample size for the intervention group, we conducted a power analysis using GPower 3.1 software. The analysis was based on an expected medium effect size of 0.5, a significance level (alpha) of 0.05, and a desired power of 0.80. The results indicated that a sample size of 27 participants was required for the intervention group to detect a statistically significant change in sedentary time between baseline and the 6-week follow-up. A total of 90 medical students were initially recruited for the study to account for potential dropouts and incomplete recalls. Of these, 13 participants were excluded due to incomplete recalls, resulting in a final sample size of 77 participants. These participants were then assigned to either the SF group (*n* = 37) or the C group (*n* = 40) using stratified random sampling.

Descriptive statistics were computed for variables to summarize the baseline characteristics of the participants. Means and standard deviations were calculated for continuous variables, while frequencies and percentages were calculated for categorical variables.

The Kolmogorov–Smirnov test revealed that the data were normally distributed. To further explore within-group changes, paired t-tests were conducted for each group to compare sedentary time at baseline and post-intervention. A two-way repeated measures ANOVA was used to examine whether the change in the sedentary time from the baseline to post-intervention between the two groups was due to the intervention.

## 3. Results

### 3.1. Participant Characteristics

Supplementary Table S1 shows the characteristics of the participants. Of the 77 participants, 57.1% were male (*n* = 44) and 42.9% were female (*n* = 33). Participants were evenly distributed across academic years, ranging from the second to the sixth year. The proportions of their body mass index (BMI) categories were 26.0% underweight, 39.0% normal weight, and 35.0% overweight/obese. Table 1 presents the participants’ characteristics according to their treatment groups. Chi-square analysis revealed no significant differences in demographic characteristics between the C and SF groups.

### 3.2. Sedentary Time

Supplementary Figure S1 displays the overall sedentary time of all participants, as well as stratified by gender, study year, and BMI. The average sedentary time was 12.8 h (SE = 0.43). The data indicate variations in sedentary time across different demographics. While males exhibited slightly higher sedentary times compared to females, this difference was not statistically significant. BMI also influenced sedentary time, with overweight individuals displaying a trend toward lower sedentary times compared to normal-weight and underweight participants.

Supplementary Figure S2 presents a breakdown of sedentary time across different activity domains. This figure reveals that participants primarily spent their sedentary time in the school/education and leisure domains, with a higher portion of sedentary time dedicated to activities associated with studying and coursework.

### 3.3. Intervention Effect on Sedentary Time

**Figure 2:**
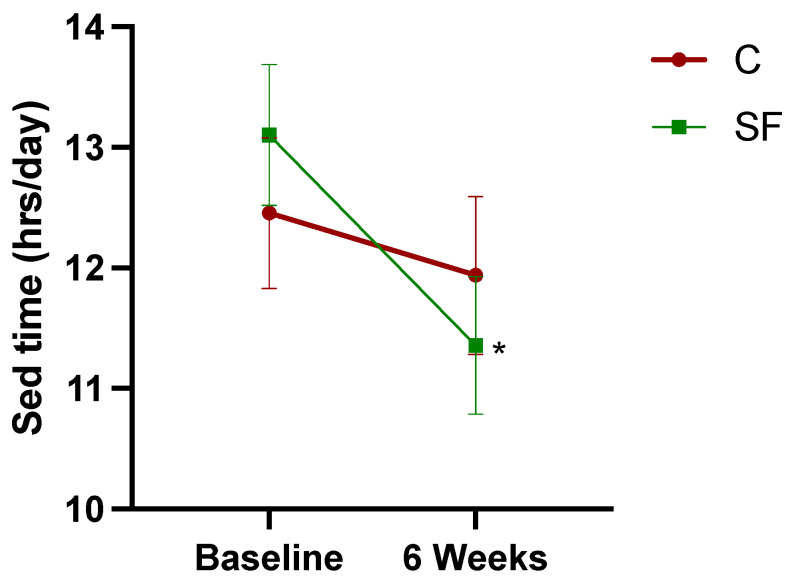
Sedentary time changes from baseline to 6 weeks post-intervention. Data points represent mean sedentary time, with error bars indicating the standard error. The asterisk (*) denotes a statistically significant pairwise comparison of pre/post-intervention sedentary time within the intervention group, with a significance level of *p* < 0.05.

Paired t-tests indicated that the SF group experienced a significant reduction in sedentary time from baseline to post-intervention (*p* = 0.01). In contrast, the C group did not show a significant change (*p* = 0.49) (Figure 2). A two-way repeated measures ANOVA revealed a significant main effect of time, F (1, 75) = 5.09, *p* = 0.03, indicating a decrease in sedentary time across all participants. However, neither the main effect of group F (1, 75) = 0.01, *p* = 0.92, nor the interaction effect F (1, 75) = 1.59, *p* = 0.21 were significant.

**Figure 3:**
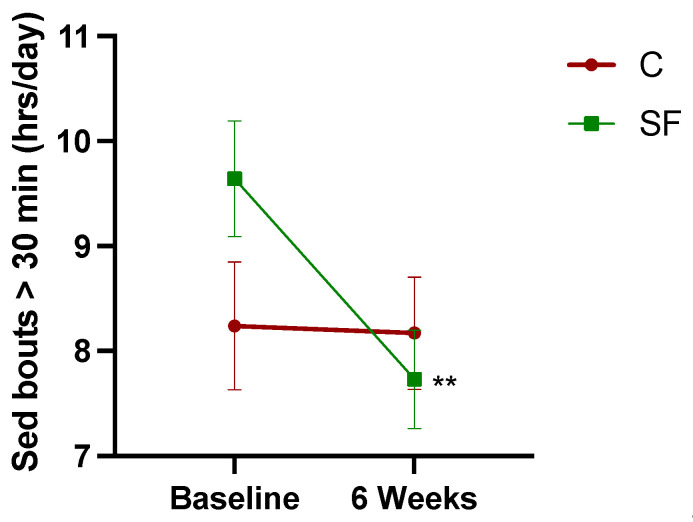
Prolonged (>30 min) sedentary time changes from baseline to 6 weeks post-intervention. Data points represent mean prolonged sedentary time, with error bars indicating the standard error. The asterisk (**) denotes a statistically significant pairwise comparison of pre/post-intervention sedentary time within the intervention group, with a significance level of *p* < 0.01.

Figure 3 illustrates the effect of the intervention on prolonged sedentary time. The paired t-test revealed that the SF group had a significant reduction in its prolonged sedentary time (*p* = 0.001), whereas the C group showed no significant change (*p* = 0.92). The repeated measures ANOVA revealed a significant main effect of time F (1, 75) = 4.05, *p* = 0.04. The main effect of the group was not significant, F (1, 75) = 0.696, *p* = 0.41. While there was a trend toward the intervention being effective in reducing prolonged sedentary bouts, the interaction effect did not reach statistical significance, F (1, 75) = 3.499, *p* = 0.06.

### 3.4. Physical Activity

Supplementary Figure S3 shows the overall PA levels and the PA of participants according to their gender, study year, and BMI. The overall PA was 3 h (SE = 0.31). Females engaged in significantly more PA than males (*p* = 0.03). Supplementary Figure S4 illustrates the PA levels across various domains. The data indicate that the household and school/education were the primary domains where participants engaged in PA.

### 3.5. Intervention Effects on Physical Activity

**Figure 4:**
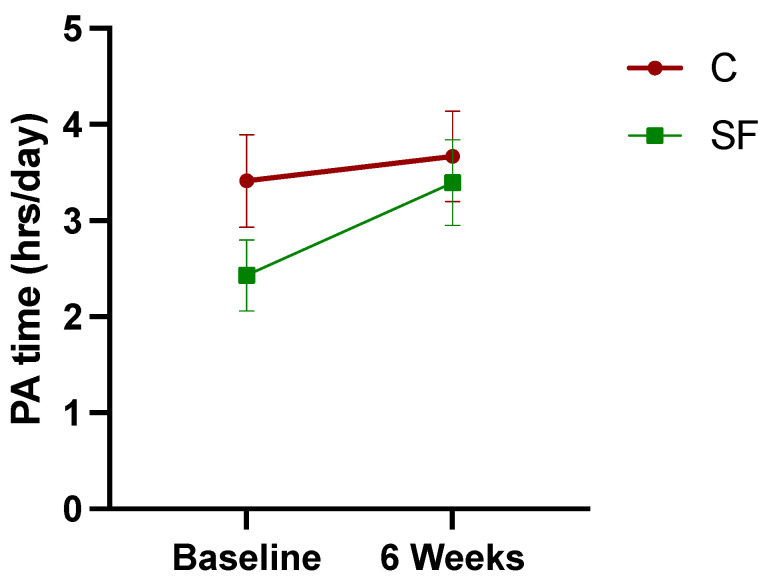
Changes in PA hours from baseline to 6 weeks post-intervention. Data points represent mean PA time with error bars indicating the standard error.

Although there was a trend toward increased PA hours in the SF group, paired t-tests revealed no significant changes in PA levels for either the SF group (*p* = 0.143) or the C group (*p* = 0.709). (Figure 4). A two-way repeated measures ANOVA revealed no significant main effects of time, F (1, 75) = 1.672, *p* = 0.200 or group, F (1, 75) = 2.223, *p* = 0.140, and no significant interaction effect, F (1, 75) = 0.565, *p* = 0.455.

### 3.6. Changes in Sedentary Time and Physical Activity According to Domains

**Figure 5:**
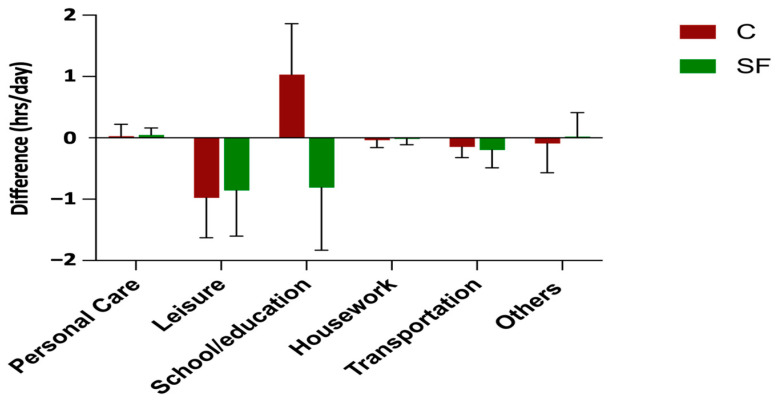
Changes in sedentary time across various domains for C and SF groups.

**Figure 6:**
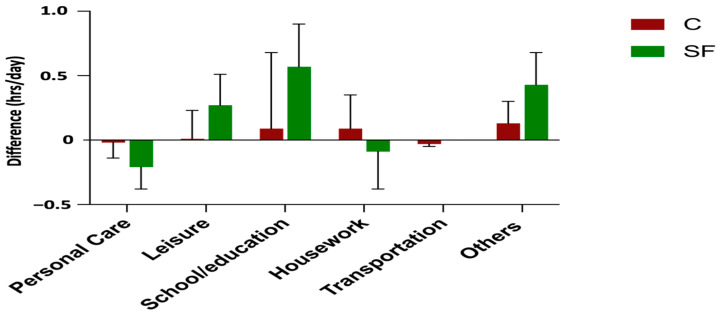
Changes in PA hours across various domains for C and SF groups.

We then examined which domains contributed to decreased sedentary time in the SF group. Figure 5 and Figure 6 illustrate the changes in sedentary time and PA hours across different domains. The school/education domain appears to be the primary contributor to the reduction in sedentary time within the SF group. This domain also contributed most to the trend of increased PA in the SF group, followed by leisure activities and activities in other domains such as spiritual activities and private time.

## 4. Discussion

This study evaluated the effectiveness of a mobile app intervention in reducing sedentary time among medical students through regular prompts to interrupt extended periods of inactivity. Our findings revealed that the intervention decreased overall sedentary time and prolonged sedentary bouts, particularly in the school domain. While the control group showed a trend toward reduced sedentary time, likely due to the small amount of education received on the adverse effects of SB, participants receiving feedback through mobile app vibrations experienced significant reductions in both total sedentary time and time spent in prolonged sedentary bouts.

Previous studies have shown that prompts and reminders can effectively break up SB [21,22,23]. For instance, workplace interventions using computer prompts have successfully reduced SB [24,25]. Consistent with these findings, our study revealed that a mobile app delivering regular prompts to break SB reduced the total sedentary time and prolonged sedentary bouts. Unlike previous interventions that focused on specific domains, such as work or school hours [26,27], our study targeted SB across multiple domains. Our results indicated that the school/education domain was the primary contributor to the reduction in sedentary time within the intervention group. This finding may seem paradoxical given that university students often spend extended periods sitting during lectures. However, university students typically have more autonomy and flexibility in their schedules compared to other populations, allowing them to incorporate movement between classes or during breaks [28]. The mobile app prompts may have encouraged students to make more active choices during these times, such as standing or walking to their next class instead of sitting. Furthermore, the school domain encompasses not only time spent in lectures but also other academic activities, such as studying in libraries or working on group projects. The intervention may have encouraged students to incorporate more standing or movement into these activities, such as taking short activity breaks while studying. Given that the school/education domain was also the major contributor to overall SB, focusing interventions on this setting has a high potential for reducing SB.

While the intervention reduced total sedentary time and prolonged bouts of SB, it did not promote PA. Similar findings have been observed in previous studies, where sedentary feedback from wearable devices reduced sedentary time accumulated in long bouts (>30 min) but did not increase PA [19]. This suggests that participants may have replaced prolonged sitting with standing or light movement rather than engaging in moderate or vigorous PA. Several factors likely contributed to this; first, our intervention (30 min interval vibration feedback) was primarily designed as a reminder tool to break up sedentary time rather than to initiate MVPA. Second, medical students face significant time constraints and academic pressures, which may limit their perceived ability to engage in more intense PA during study or work hours, especially given that our results showed that students spent most of their sedentary time at school. In addition, the physical environment of academic settings, often designed for seated work, may also contribute to this pattern by providing limited opportunities for more active behaviors [29]. 

While our results indicated a disconnection between SB and PA in terms of the intervention’s effect, future interventions should aim to address both behaviors simultaneously. Understanding the barriers that prevent medical students from increasing their PA, even when they reduce their sedentary time, is crucial for designing effective interventions. Vandelanotte et al. demonstrated that real-time feedback is an effective tool for increasing PA when used with another support system, such as detailed web-based feedback and trainer support [30]. This suggests that future medical student interventions could benefit from combining real-time feedback with an additional support system with tailored PA feedback and advice. Furthermore, real-time vibration feedback can be integrated with behavior intervention strategies that can be implemented on a large scale at a low cost. For example, utilizing theoretical frameworks such as the Health Action Process Approach could enhance health behaviors such as PA in part via developing action plans and coping strategies to sustain these behaviors over time [16].

Although most epidemiological evidence indicates that prolonged sitting is associated with a higher risk of cardiovascular disease and all-cause mortality, independent of moderate-to-vigorous PA [31,32,33], there is evidence that breaking up sitting time can improve cardiac and metabolic parameters [8]. While participants in our study were young and likely free from psychopathological issues, previous research has demonstrated that interrupting sedentary time has a positive effect on mood [19]. This suggests that our findings may also confer additional health benefits such as improved mood and metabolic health.

Our results indicate a gender difference in reported PA, with females demonstrating higher levels of PA than males. This finding contrasts with previous studies, which generally show that males tend to be more physically active [34,35]. One possible explanation for this discrepancy is the difference in the instruments used to assess PA. Nevertheless, the higher PA in females in our study should be interpreted with caution due to our small sample size. Furthermore, it is important to note that this gender difference reflects overall PA levels and not PA in response to the intervention. Further domain analysis revealed that females’ PA at school is higher than males’. This may be attributed to females’ higher perception of the health risks associated with inactivity [36,37], prompting them to adhere more strictly to the sedentary feedback prompts and engage in more PA. Future research with larger sample sizes and objective assessment methods could help clarify these gender-related patterns in PA among medical students and explore the underlying factors contributing to these differences. While these gender differences were not the primary focus of our study, they provide important context for future research in this area.

While questionnaire-based estimates for habitual PA are useful, they may be challenging for participants to complete thoroughly, making it difficult to obtain a comprehensive assessment of daily activities. For instance, recalling and reporting individual PA over the past week may be cognitively demanding. Although these questionnaires provide details regarding exercise and recreation, they often offer limited insight into other domains like household activities and sedentary behavior. The ACT24 tool can overcome some of the limitations of questionnaire-based assessments (e.g., the International Physical Activity Questionnaire), including recall bias and the incomplete coverage of activity domains. The domain-specific activity information generated from the ACT24 can be used to target domains where there is potential for reducing SB and promoting PA. For example, our results show that the school/education and leisure domains were the two areas where medical students engaged in the most SB, constituting almost 70% of their sedentary time. This reinforces the importance of reducing SB in these settings using established strategies [38].

While the repeated measures ANOVA provides a more robust analysis of our data than the paired t-test, especially given the longitudinal design of the study, with multiple measurements, the interaction effect was not statistically different. This suggests that the change in sedentary time over the intervention six-week period was not different between the C and SF groups. Several factors may contribute to this outcome: first, the small sample size and high variability may not provide sufficient power to detect the small effect of the intervention. Second, while the effect size in the experimental groups appears to be moderate (about a 1.78 h reduction), when compared to the control group’s minimal change (0.4 h), the overall effect size between groups is smaller. In addition, the post-intervention reduction in sedentary time in the C group, possibly due to the small amount of education received on the adverse effects of sedentary behavior, may have diluted the effect of the intervention. Furthermore, the relatively short duration of our study might not have been enough to capture the full trajectory of the change in sedentary time in response to the intervention.

While there are no specific recommendations for sedentary time in healthy adults, the intervention group demonstrated a substantial decrease in total sedentary time by approximately 13%, compared to a 3% decrease in the control group. Similarly, for prolonged sedentary bouts, the intervention group exhibited a notable reduction of approximately 19%, whereas the control group showed a minimal decrease of about 1%. These percentage decreases suggest that this intervention may potentially have a meaningful clinical impact on the health of university students. Previous studies have indicated that even modest reductions in sedentary time can have positive health outcomes, such as improved metabolic health and a decreased risk of cardiovascular disease [8,39]. Reallocating two hours per day from sitting time to standing or stepping is associated with reductions in body mass index, waist circumference, triglyceride, and plasma glucose in adults [40]. Thus, a two-hour reduction in sitting time along with an increase in standing and stepping during school may potentially result in beneficial health effects, especially if maintained over a long time.

This study has several limitations that should be considered. First, it relied on self-reported measures of sedentary time and PA, which are susceptible to recall bias and may not accurately reflect actual behavior. Second, there was no minimum sedentary time criterion for participant inclusion, which may have contributed to the variability in baseline sedentary time. Additionally, our study relied on participant recalls conducted for only one day at baseline and one day at the six-week follow-up. This limited sampling may not fully capture typical behavior due to inherent day-to-day variability in individual sedentary and PA patterns [41]. Moreover, we did not collect data on participants’ past sports involvement or prior physical activity, limiting our ability to assess how previous PA engagement may have influenced intervention responsiveness. Moreover, the intervention was short in duration and conducted among medical students during a transient stage of their lives.

## 5. Conclusions

In conclusion, our findings highlight the potential of mhealth to promote behavior changes and offer a promising strategy for reducing sedentary behavior, especially in educational settings. Future research may consider combining SB interruptions with structured incentives to promote PA. One strategy is an integrated campus incentive; for instance, future studies may consider utilizing wearable devices to track sedentary breaks and MVPA, which could be linked to a reward system integrated with campus services (e.g., bookstore discounts, meal plan bonuses). Another strategy is the loss-framed financial incentive approach [42], in which allocated finance could be lost if PA goals are not met. Future studies may also address the long-term maintenance (i.e., 12 months and beyond) of behavioral change (reduced SB and enhanced PA), and strategies that may help maintain PA once intervention ends.

## Figures and Tables

**Table 1:** Participants’ characteristics according to the intervention.

		C		SF		*p*
		*n*	%	*n*	%	
Gender	Male	22	50.5	22	50.5	0.70
	Female	18	54.5	15	45.5	
Year	2nd year	11	61.1	7	38.9	0.61
	3rd year	10	52.6	9	47.4	
	4th year	6	37.5	10	62.5	
	5th year	6	46.2	7	53.8	
	6th year	7	63.6	4	36.4	
BMI	Underweight	10	50.0	10	50.5	0.80
	Normal weight	17	56.7	13	43.3	
	Overweight/obese	13	48.1	14	51.9	

BMI: Body mass index.

## Data Availability

The data that support the findings of this study are available from the corresponding author upon reasonable request.

## Supplementary Materials

The following supporting information can be downloaded at: https://www.mdpi.com/article/10.3390/healthcare12212133/s1, Table S1: Participants’ characteristics; Figure S1: title; Figure S1: Sedentary time for all participants and according to gender, study year and BMI; Figure S2: Sedentary time according to major activity domains; Figure S3: Physical activity hours per day for all participants and according to gender, study year and BMI; Figure S4: PA hours across various domains.
