# Supplementary material for: Real-Time Vibration Feedback from a Smartphone Application Reduces Sedentary Time but Does Not Increase Physical Activity Among Medical Students
Source: Healthcare (Basel). 2024 Oct 26;12(21):2133. doi: 10.3390/healthcare12212133 (PMC11545671; doi:10.3390/healthcare12212133)
Supplement: Supplementary file 1 [file healthcare-12-02133-s001.zip › healthcare-3248331-supplementary.pdf]

Supplementary Materials

***Supplementary Table S1**. Participants' characteristics*

		N (77)	%
Gender	Male	44	57.1
	Female	33	42.9
Year	2nd year	18	23.4
	3rd year	19	24.7
	4th year	16	20.8
	5th year	13	16.9
	6th year	11	14.3
BMI	Underweight	20	26.0
	Normal weight	30	39.0
	Overweight/Obese	27	35.0

BMI: Body mass Index

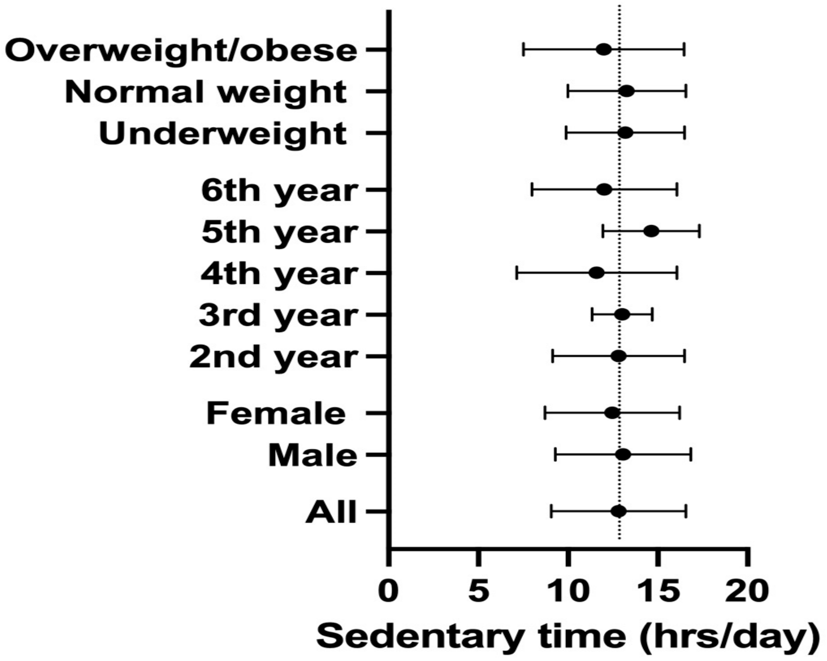

***Supplementary Figure S1.** Sedentary time for all participants and according to gender, study year and BMI*

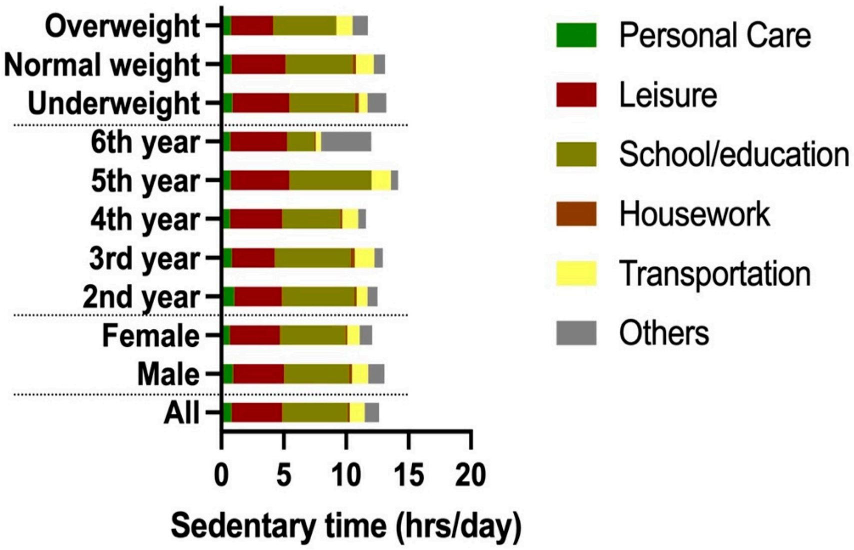

Supplementary Figure S2. *Sedentary time according to major activity domains*

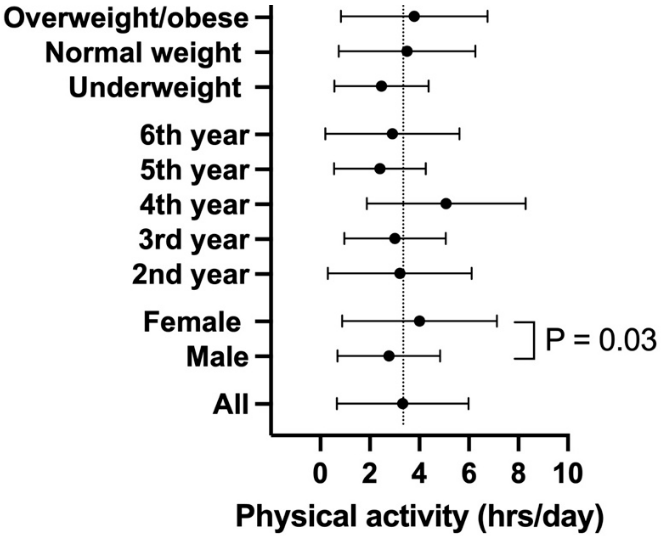

Supplemntary Figure S3. *Physical activity hours per day for all participants and according to gender, study year and BMI.*

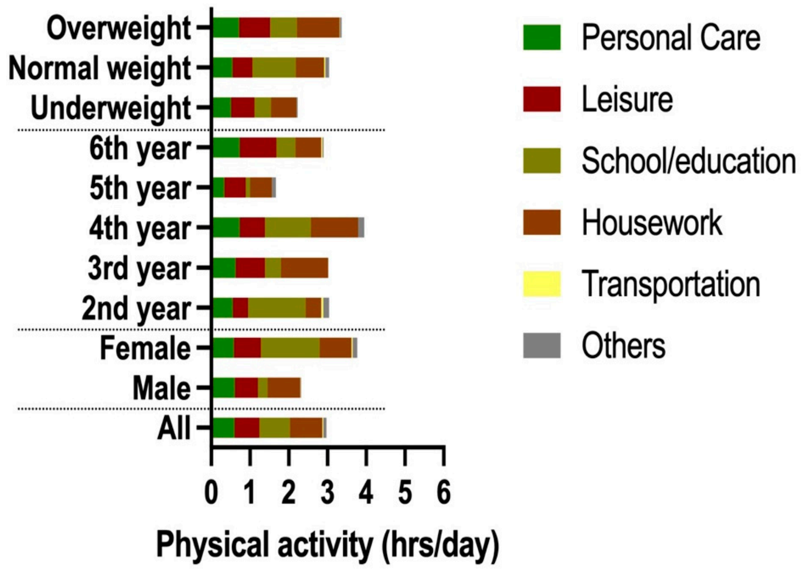

Supplementary Figure S4: PA hours across various domains.
